# A Comprehensive Review of Coronavirus Non-Structure Protein 6 on Structure, Functions, Mechanisms and Its Implications for Antiviral Research

**DOI:** 10.3390/v18070721

**Published:** 2026-06-30

**Authors:** Yingzhe Yu, Weimei He, Xiaohui Geng, Yulong He, Huapeng Feng, Jian Chen, Jianhong Shu

**Affiliations:** 1 College of Life Sciences and Medicine, Zhejiang Sci-Tech University, Hangzhou 310018, China; yyz51127@163.com (Y.Y.);; 2 Hangzhou ISEEVAX Medical Sciences & Technology, Co., Ltd., Hangzhou 310018, China

**Keywords:** coronavirus, NSP6, double-membrane vesicle, autophagy, innate immunity, antiviral target

## Abstract

Coronaviruses encode a variety of non-structural proteins (NSPs) that collectively mediate viral genome replication, transcription and remodeling of the host cellular microenvironment. As a highly conserved transmembrane protein, non-structural protein 6 (NSP6) predominantly localizes to the endoplasmic reticulum. Through interactions with other viral proteins and host factors, NSP6 participates in multiple pivotal processes, including the formation and stabilization of double-membrane vesicles (DMVs), reprogramming of lipid metabolism, blockade of autophagic flux, and evasion of innate immunity. Recent advances in structural biology and research on virus–host interactions have further elucidated the essential roles of NSP6 throughout the viral life cycle. Mutations in NSP6 are closely associated with viral adaptability, transmissibility and pathogenicity. Herein, we comprehensively review the latest advances on the molecular structure, biological functions and mutation hotspots of coronavirus NSP6, as well as its implications for antiviral research. This review aims to provide a theoretical basis for further dissecting the pathogenic mechanisms of coronaviruses and developing broad-spectrum antiviral drugs.

## 1. Introduction

Coronaviruses (CoVs) are enveloped viruses with positive-sense single-stranded RNA genomes. They infect humans and a wide range of animals, causing diseases of the respiratory, digestive and nervous systems [1,2]. In 2014, the International Committee on Taxonomy of Viruses (ICTV) classified the family Coronaviridae into four genera: *Alphacoronavirus*, *Betacoronavirus*, *Gammacoronavirus* and *Deltacoronavirus* [3]. Coronavirus particles are approximately 80–120 nm in diameter, consisting of an envelope and an internal ribonucleoprotein complex. The viral genome comprises genes encoding non-structural proteins, structural proteins and accessory proteins, as well as untranslated regions at both termini. The 5′ end of the genome contains a methylated cap structure, while the 3′ end possesses a poly(A) tail. With a full length of 27–32 kb, coronaviruses harbor the largest genomes among all known RNA viruses [4,5]. The 5′ two-thirds of the coronavirus genome encodes two replicase polyproteins, pp1a and pp1ab. The production of pp1ab relies on a programmed ribosomal-1 frameshift that occurs at the end of the pp1a coding region. These two polyproteins are subsequently cleaved by viral proteases to generate 16 non-structural proteins (NSP1-NSP16) [6,7,8,9], collectively assemble into the viral replication-transcription complex (RTC), which acts as the core machinery for viral replication in host cells [10,11,12,13]. Among them, NSP3, NSP4 and NSP6 are key transmembrane proteins responsible for endomembrane remodeling. They remodel the host endoplasmic reticulum to form double-membrane vesicles (DMVs), the protected compartments for viral replication [14]. Emerging outbreaks caused by zoonotic coronaviruses, including severe acute respiratory syndrome coronavirus (SARS-CoV), a member of the species *Betacoronavirus pandemicum*, in 2003 [15], *Betacoronavirus cameli* (MERS-CoV) in 2012 [16], and severe acute respiratory syndrome coronavirus 2 (SARS-CoV-2), also classified under *Betacoronavirus pandemicum*, since 2019 [17], have demonstrated the persistent threat of coronaviruses to global public health. As a functionally versatile transmembrane protein associated with viral replication, NSP6 serves as a crucial component for DMV biogenesis. It also comprehensively remodels the host cellular environment to favor viral survival by regulating autophagy [18], lipid metabolism [19,20], lysosomal function [21] and innate immune signaling pathways [22,23]. Unlike the highly variable spike (S) protein, NSP6 exhibits relatively high sequence conservation across coronaviruses [24], making it an attractive target for the development of broad-spectrum anti-coronavirus therapeutics. Here, we review the diverse structure and biological functions of NSP6, aiming to provide new insights for fundamental research and control strategies against coronaviruses.

## 2. Molecular Characteristics and Structural Features of NSP6

### 2.1. Gene Location and Fundamental Properties of Proteins

**Figure 1:**
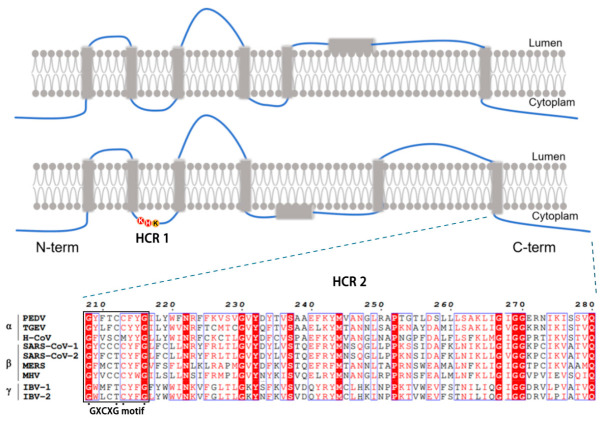
Two topological structure model predictions of coronavirus NSP6 (**upper panel**) and alignment analysis of the highly conserved C-terminal region (**lower panel**). The KHK motif in the highly conserved region (HCR) 1 and the GXCXG motif, supposed to be palmitoylated, in HCR 2 are particularly highly conserved and exposed to the cytosol.

NSP6 is encoded by the ORF1a of coronaviruses and liberated from the polyprotein precursor via sequential cleavage by papain-like protease (PLpro) and 3C-like protease (3CLpro) [25]. The nsp6 gene accounts for approximately 1% of the viral genome, and the number of amino acid residues of NSP6 varies among different coronaviruses. Specifically, NSP6 consists of around 300 amino acids in SARS-CoV-1, 290 in SARS-CoV-2, 292 in MERS-CoV, 296 in *Alphacoronavirus porci* (PEDV), 287 in *Betacoronavirus muris* (MHV), and 306 in *Gammacoronavirus galli* (IBV). Depending on viral species, NSP6 is folded into multiple transmembrane domains (TM), including six to seven conserved helical domains (Figure 1) [26]. As a highly hydrophobic endoplasmic reticulum transmembrane protein, NSP6 anchors itself to the membrane of intracellular DMVs. For instance, SARS-CoV-2 NSP6 predominantly localizes to the endoplasmic reticulum and perinuclear region [27]. Multiple sequence alignment of coronavirus NSP6 reveals two highly conserved regions (HCR), including the KHK motif and C-terminus, both located in the cytosol, reflecting its evolutionary stability. The transmembrane regions and topological structures of NSP6 are highly conserved across *Alphacoronavirus*, *Betacoronavirus*, and *Gammacoronavirus*, which demonstrates that the functions of NSP6 are indispensable for viral replication.

### 2.2. Topological Structure

Coronavirus NSP6 is an endoplasmic reticulum (ER) transmembrane protein. Its core topological characteristics were first identified in classic model coronaviruses, including MHV and IBV, using molecular biological approaches such as glycosylation tagging and protease protection assays and further validated in SARS-CoV and SARS-CoV-2 in subsequent studies [24,28,29,30]. Bioinformatic analyses generally predict that NSP6 harbors seven hydrophobic domains, among which only six function as transmembrane helices. Starting from the cytoplasmic N-terminus, these six transmembrane helices traverse the ER lipid bilayer six times, with the C-terminus remaining on the cytoplasmic side without spanning the membrane. In brief, both the N- and C-termini of NSP6 are fully exposed to the cytosol and do not extend into the ER lumen. The C-terminal region of NSP6 forms a large cytoplasmic tail consisting of approximately 60–80 amino acids. This region contains a conserved hydrophobic segment and membrane-associated element (MAE) [24], serving as a critical interface for interactions with host proteins and other non-structural proteins.

Taking SARS-CoV-2 NSP6 as an example, residues 1–11 at the N-terminus are entirely localized in the cytosol. This region harbors the cleavage site for NSP5, which must face the cytoplasm to ensure protein maturation [30]. The transmembrane helices TM1-TM6 (residues 12–230) traverse the membrane six times, forming short loops exposed to the ER lumen and cytosol respectively. These transmembrane domains serve as the structural foundation for DMV biogenesis and are also essential for ER anchoring, membrane curvature induction and protein dimerization. The central non-transmembrane region (residues 91–112) resides exclusively within the ER lumen. Though not involved in membrane spanning, it acts as a vital interface for protein–protein interactions [31]. The C-terminal cytoplasmic domain (residues 231–290) is a conserved hydrophobic segment located entirely in the cytosol. Containing the MAE, this domain mainly recruits lipid droplets and components of endoplasmic reticulum-associated degradation (ERAD) [20], as well as autophagy-related proteins, thereby regulating viral replication and immune evasion. The topological organization [29] and biological functions [32] of distinct NSP6 regions are highly conserved across other coronaviruses, including PEDV, MHV and IBV.

The high conservation of the topological architecture and biological functions of NSP6 among *Alphacoronaviruses* (e.g., PEDV), *Betacoronaviruses* (e.g., SARS-CoV and MHV) and *Gammacoronaviruses* (e.g., IBV) indicates that NSP6 is a core replication protein retained throughout coronavirus evolution. Its conserved topological conformation is indispensable for viruses to establish replication organelles and complete the replication cycle, which highlights the potential of NSP6 as a broad-spectrum target for anti-coronavirus drug development.

## 3. Core Biological Functions of NSP6

**Figure 2:**
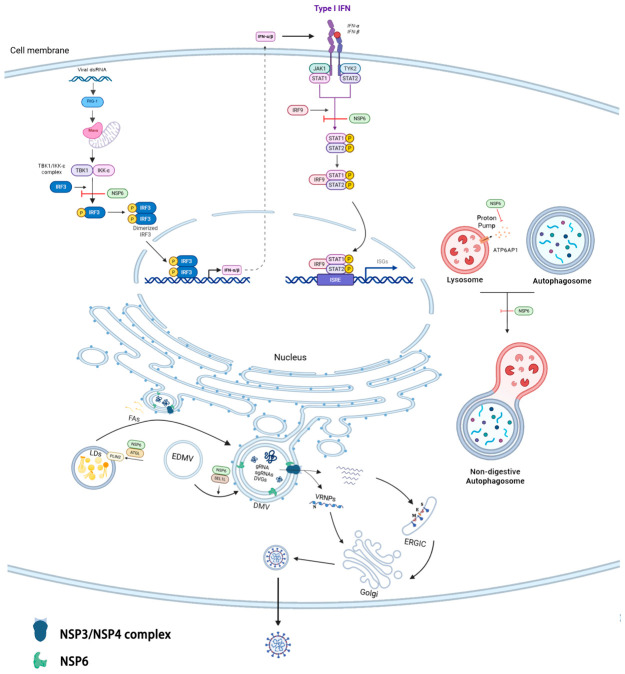
Mechanism diagram of coronavirus NSP6 function. The coronavirus NSP6, in collaboration with NSP3 and NSP4, undergoes oligomerization, inducing curvature and fusion of the endoplasmic reticulum membrane to form DMVs. NSP6 can anchor lipid droplets, hijack endoplasmic reticulum lipids to interfere with the ERAD pathway in degrading PLIN2, and accelerate lipid utilization, thereby regulating lipid metabolism and the ERAD pathway. Additionally, NSP6 inhibits the MLN1/ATP6AP1 proton pumps, blocking autophagy flux. NSP6 binds to TBK1/IKKε, preventing IRF3 phosphorylation and nuclear translocation and suppressing IFN-I expression, thus antagonizing the host cell’s innate immune response. Created in BioRender. Yu, Y. (2026) https://BioRender.com/zehsadq (accessed on 16 June 2026).

As a core scaffold protein of the coronavirus replication complex, NSP6 plays an essential role in the viral life cycle (Figure 2 and Table 1). It not only drives the formation of viral replication organelles and modulates host innate immunity but also mediates autophagy and organelle reprogramming to promote the persistent survival and proliferation of viruses in host cells.

### 3.1. Participation in the Formation of DMVs

Positive-sense single-stranded RNA viruses commonly remodel intracellular membranes to facilitate viral replication [36,37]. Coronaviruses generate specialized replication organelles termed double-membrane vesicles (DMVs) to serve as platforms for viral replication. These structures efficiently recruit viral proteins and associated host factors and shield viral RNA from recognition by the host immune system. Derived from the endoplasmic reticulum during infection, DMVs are the primary sites for viral genome replication. Viral non-structural proteins together with host factors constitute the microenvironment of the coronavirus replication-transcription complex (RTC) [38]. DMVs further provide a favorable milieu and protective effects for RTC-mediated replication [13,39,40,41,42,43].

Given the high complexity and dynamic nature of replication-associated organelles and DMVs, their biogenesis mechanisms remain incompletely elucidated. Studies have demonstrated that NSP3 and NSP4 represent the minimal viral components required to initiate the formation of DMV pore complexes in MHV and SARS-CoV-2 [44]. Possessing membrane proliferation activity, NSP6 induces perinuclear vesicles around the microtubule-organizing center and markedly increases the number of DMVs generated by NSP3 and NSP4. Deletion or mutation of NSP6 drastically reduces the abundance and structural integrity of DMVs, thereby directly impairing viral replication [14,20]. Cryo-electron microscopy (cryo-EM) analyses reveal that DMVs adopt a double-membrane architecture and localize to the cytoplasmic side at the periphery of the perinuclear endoplasmic reticulum. Cryo-electron tomography combined with subtomogram averaging indicates that the NSP3-NSP4 pore complex is composed of 12 copies each of NSP3 and NSP4, which assemble into four concentric stacked hexameric rings. This miniature nuclear pore-like complex is presumably involved in mediating RNA translocation [45]. In SARS-CoV-2, DMV formation is triggered by NSP3 and NSP4. Meanwhile, NSP6 tethers to the ER membrane and establishes membrane contact sites via homo-oligomerization and amphipathic helix zippers. These three transmembrane proteins act synergistically to drive DMV biogenesis. Specifically, NSP3 and NSP4 form a zipper-like structure to bring ER membrane bilayers into close proximity, while NSP6 facilitates membrane curvature and vesicle budding and stabilizes DMV structures. Collectively, they assemble into a large complex with six-fold symmetry that constitutes the vesicle pore [46]. DMVs serve as a physical barrier to protect viral RNA from degradation. The vesicle pores are proposed to mediate the export of newly replicated viral RNA [42,47,48].

### 3.2. Regulation in Lipid Metabolism and the ERAD Pathway

Lipid droplets (LDs) are essential organelles that store neutral lipids, including triacylglycerols (TGs) and cholesterol esters, and play a vital role in cellular lipid metabolism and energy homeostasis [49]. Fatty acids (FAs) sequestered within the LD core in the form of TGs are recycled mainly through lipophagy and lipolysis [50,51].

Recent studies have revealed that NSP6 hijacks the host endoplasmic reticulum-associated degradation (ERAD) pathway and lipid metabolism. It anchors DMVs to the surface of LDs via its transmembrane domains [20]. Confocal microscopy and immunoelectron microscopy analyses have demonstrated the colocalization of NSP6 with the LD marker PLIN2 and the neutral lipid dye Bodipy493/503. The amphipathic helix located within the 80 amino acids at the C-terminus of NSP6 mediates its binding to the LD membrane. Sucrose density gradient centrifugation further verified the enrichment of NSP6 in purified LD fractions. These findings indicate that NSP6 can access LDs, laying a foundation for the subsequent hijacking of lipid metabolic pathways. Expression of NSP6 markedly reduces the number of LDs and cellular TG content. This process is independent of lipophagy and instead relies on the proteasomal degradation of the LD coat protein PLIN2. Inhibition of adipose triglyceride lipase (ATGL) reverses NSP6-induced LD degradation. During the early stage of coronavirus infection, the levels of TGs and PLIN2 rise transiently and then decline sharply, suggesting that the virus first triggers LD biogenesis and subsequently promotes lipolysis. In this process, the region spanning residues 1–175 at the N-terminus of NSP6 interacts with core ERAD components such as EDEM1, OS9 and SEL1L, thereby targeting PLIN2 for degradation. The loss of PLIN2 exposes TGs in LDs to lipases, including ATGL, leading to the release of free FAs [20,52]. The liberated FAs are transported to DMV membranes to serve as phospholipid precursors for membrane synthesis and support DMV expansion.

Moreover, NSP6 facilitates DMV biogenesis by recruiting ERAD-derived vesicles. Upon the completion of misfolded protein degradation, the ERAD complex disassembles and generates EDEM1/OS9-positive vesicles (EDMVs). By binding to ERAD components including SEL1L, NSP6 captures EDMVs and delivers them to DMVs [53]. Carrying preformed membrane structures composed of phospholipids and cholesterol, EDMVs fuse directly with DMVs to accelerate membrane growth. Sucrose density gradient centrifugation shows that NSP6 expression leads to the co-enrichment of EDMV components with DMVs. Knockdown of EDEM1 or OS9 suppresses coronavirus replication, confirming that EDMVs are a critical source of membrane materials for DMVs. In this manner, NSP6 directly supplies preassembled membrane constituents for DMV expansion. The viral accessory protein orf3a also exerts a promotive effect on these biological processes [19].

The mechanisms by which NSP6 regulates lipid metabolism and the ERAD pathway are highly conserved across the Coronaviridae family. These processes have been systematically validated in *Betacoronaviruses*, including SARS-CoV, SARS-CoV-2 and MHV. Additionally, studies on *Alphacoronaviruses* such as PEDV have confirmed that NSP6 localizes to the ER-LD interface and induces lipolysis via analogous pathways to supply lipid materials for DMVs [53]. These findings further demonstrate the evolutionary conservation of NSP6 as a core regulatory protein for coronavirus replication. Overall, NSP6 drives DMV growth in a dual manner through the spatiotemporal coupling of the ERAD machinery and reprogramming of lipid metabolism. At the early stage of viral infection, NSP6 promotes the release of fatty acids from LDs to provide lipid precursors for DMV biogenesis. During the phase of robust viral replication, NSP6 recruits ERAD-derived vesicles to deliver membrane components for DMV expansion, thereby sustaining efficient viral replication.

### 3.3. Modulating the Autophagy Pathway to Facilitate Viral Replication

Autophagy as a cellular self-defense mechanism. On one hand, it delivers viruses and viral proteins to lysosomes for degradation, eliminating invading pathogens and mediating host innate immune defense. On the other hand, pathogens can also exploit autophagy to enhance their infectivity [54,55]. Cottam et al. first demonstrated that SARS-CoV NSP6 induces autophagosome formation via the omegasome pathway. The resulting small-sized autophagosomes serve as double-membrane vesicle platforms to support viral replication, and this function is highly conserved among coronaviruses [56]. As a key modulator of host autophagy, NSP6 binds to the autophagy regulator Beclin1 and increases its phosphorylation level, thereby initiating autophagy [33,57]. Moreover, NSP6 interacts with mucolipin 1 (MLN1) to impair efficient degradation by autolysosomes [21]. Collectively, these effects lead to autophagosome accumulation and blocked autophagic flux, which ultimately benefits viral replication.

NSP6 is capable of restricting autophagosome expansion [58]. Studies have revealed that IBV NSP6 triggers autophagosome biogenesis originating from the endoplasmic reticulum, and the resulting autophagosomes are smaller in diameter than those induced by nutrient starvation. Consistently, cells infected with IBV, as well as cells expressing NSP6 from MHV and SARS-CoV, produce small-sized autophagosomes. A similar phenotype is also observed for NSP5, NSP6 and NSP7 of porcine reproductive and respiratory syndrome virus (PRRSV). NSP6 likely restricts autophagosome size during omegasome formation and suppresses the expansion of autophagosomes and omegasome structures induced by starvation and Torin1 treatment. Reduced autophagosome diameter impairs the delivery of viral components to lysosomes for degradation, enabling viruses to evade clearance and facilitating viral replication.

Additionally, NSP6 inhibits lysosomal acidification, which further protects viruses within host cells. Lysosomal acidification is a critical step for autophagy, as an acidic microenvironment is essential for lysosomal degradative activity [59]. SARS-CoV-2 NSP6 interacts with components of the ATP-dependent proton pump, thereby activating the NLRP3 inflammasome and blocking lysosomal acidification [34,60]. Impaired acidification diminishes lysosomal degradation capacity and arrests autophagic flux, allowing viruses to escape lysosomal destruction and replicate efficiently. NSP6 also interferes with the formation and degradation of autolysosomes. During canonical autophagy, autophagosomes fuse with lysosomes to form autolysosomes, where engulfed cargos are degraded. SARS-CoV-2 NSP6 localizes to lysosomes and hinders autophagosome-lysosome fusion. At the late stage of autophagy, it further suppresses autolysosomal degradation via modulating MLN1, leading to defective autophagic flux and persistent viral survival and replication. This process may be associated with ER-dependent pathways, yet the detailed mechanisms remain to be elucidated [61,62,63]. Likewise, MERS-CoV NSP6 promotes the phosphorylation and activation of kinase AKT1. Activated AKT1 in turn phosphorylates and upregulates S-phase kinase-associated protein 2 (SKP2), resulting in autophagy inhibition. The abilities to induce autophagosome formation and block autophagosome-lysosome fusion are highly conserved among NSP6 proteins from diverse coronaviruses [64,65,66].

The autophagy-modulating strategies mediated by NSP6 confer substantial biological advantages for coronaviruses. On the one hand, NSP6 exploits the autophagic membrane network to expand viral replication compartments, providing sufficient space and materials to support efficient viral replication and spread. On the other hand, by blocking lysosome-mediated viral clearance, NSP6 helps viruses evade host immune surveillance and degradation, supporting persistent viral survival and proliferation in host cells.

### 3.4. Antagonizing the Innate Immune Response

Coronavirus NSP6 antagonizes host innate immune responses through multiple mechanisms, including suppression of IRF3 phosphorylation, inhibition of type I interferon responses, activation of key proteins in the autophagic degradation pathway, and disruption of the MAVS-IRF3 signaling cascade [67,68].

SARS-CoV-2 NSP6 interacts with TANK-binding kinase 1 (TBK1) and subsequently inhibits the phosphorylation of interferon regulatory factor 3 (IRF3) [69]. Phosphorylation of IRF3 is essential for the production of type I interferons. By blocking this process, NSP6 reduces type I interferon secretion, enabling the virus to evade surveillance and attack by the host innate immune system. For instance, if NSP6 suppresses IRF3 phosphorylation at the early stage of infection, the host fails to produce sufficient type I interferons in a timely manner, which facilitates viral replication and spread. Furthermore, NSP6 counteracts type I interferon responses by inhibiting IFN-β induction and downstream IFN signaling. Dual transfection assays have verified that NSP6 represses the activity of the IFN-β promoter and interferes with IFN signal transduction. As pivotal antiviral molecules of the host, type I interferons exert potent defense against viral infection. Inhibition of interferon responses by NSP6 therefore impairs host antiviral immunity [70].

NSP6 also activates core components of the autophagic degradation pathway. The TM3-4 domain (residues 81–120) of SARS-CoV-2 NSP6 triggers endoplasmic reticulum (ER) stress. NSP6 then binds to the ER chaperone HSPA5/GRP78 and activates autophagy via the EIF2AK3/PERK-EIF2A/EIF2α pathway. This process further mediates the degradation of ER-resident STING1 protein, thereby dampening type I interferon production and promoting viral innate immune evasion. In SARS-CoV-2 variants, deletions spanning residues 105–107 or 106–108 of NSP6 weaken its binding affinity to HSPA5/GRP78, as well as its capacity to induce ER stress, autophagy and STING1 degradation. Such defects compromise viral innate immune evasion, which partially accounts for the attenuated pathogenicity of variants, including Omicron [71,72,73]. In addition, NSP6 acts together with NSP13 to interact with intermediate molecules in the MAVS-IRF3 cascade and restrain IRF3 activation. These two proteins also inhibit the phosphorylation of STAT1 and STAT2, while NSP13 additionally blocks the nuclear translocation of NF-κB. The MAVS-IRF3 cascade plays a central role in innate immune signaling. Disruption of this pathway by NSP6 impedes the transmission of host immune signals and downregulates the expression of antiviral genes, ultimately establishing a favorable environment for viral survival and proliferation within host cells [74].

In summary, NSP6 binds to TBK1, suppresses the phosphorylation and nuclear translocation of IRF3, and consequently reduces the production of type I interferons. It also inhibits the activation of STAT1/STAT2 and disrupts downstream interferon signaling. Moreover, NSP6 modulates lysosomal function to indirectly regulate inflammasome activation. To date, this immune evasion activity has only been experimentally verified in *Betacoronaviruses* such as SARS-CoV-2. For *Alphacoronaviruses*, *Gammacoronaviruses* and other genera, existing studies on NSP6 primarily focus on ER membrane remodeling, DMV formation and lipid metabolism regulation. Direct experimental evidence for its involvement in innate immune evasion remains lacking and requires further investigation. The immune evasion capacity of coronavirus NSP6 enables the virus to gain an advantage at the early stage of infection.

### 3.5. NSP6 Mutations and Viral Evolution

As a highly conserved core protein involved in viral replication and immune evasion, NSP6 undergoes selectively advantageous mutations during coronavirus evolution. These alterations modulate membrane remodeling, replication efficiency and immune evasion, thereby driving viral adaptive evolution.

The deletion mutant Δ106SGF108 is one of the most representative adaptive mutations in SARS-CoV-2. This mutation has emerged independently in multiple variants, including Alpha, Beta, Gamma, Eta, Iota, Lambda and Omicron (with Omicron BA.1 carrying Δ105LSG107), representing a typical case of convergent evolution. Functional studies have demonstrated that Δ106SGF108 markedly enhances the ER zipper activity of NSP6 and improves membrane remodeling efficiency. This further facilitates DMV biogenesis and boosts viral replication, indicating a gain-of-function mutation [26,30,75]. Additionally, this deletion strengthens the antagonistic effect of NSP6 against IFN-I signaling. By intensifying the inhibition of STAT1/STAT2 phosphorylation, it enhances viral immune evasion and lays a molecular foundation for the virus to establish replicative advantages at the early stage of infection [72].

L37F is a well-characterized single nucleotide polymorphism (SNP) located within the transmembrane region of NSP6. This mutation alters the conformation and stability of the NSP6 transmembrane domain and further regulates the autophagy-lysosome pathway and inflammasome activation [21]. Mutant NSP6 exhibits reduced binding affinity for MLN1, which restores autophagic flux and decreases inflammasome activation as well as pyroptosis. This phenotype is associated with asymptomatic infection, revealing an adaptive strategy by which viruses modulate pathogenicity via mutation. Other independent adaptive point mutations such as L260F can significantly elevate viral RNA replication efficiency and pathogenicity in mice. They exert compensatory effects in variants with impaired spike-mediated entry, thus sustaining viral transmissibility [71]. The impacts of other NSP6 mutations on pathogenesis remain unclarified.

As an internal protein of viral replication organelles, NSP6 is not exposed on the viral surface [29]. Its mutations do not directly alter viral antigenicity or mediate escape from neutralizing antibodies. Instead, they improve viral fitness and transmissibility indirectly by enhancing membrane remodeling and replication efficiency, reinforcing immune evasion and regulating pathogenicity. Accordingly, NSP6 serves as a crucial genetic marker for coronavirus evolution. The independent recurrence of such mutations across diverse variants further highlights the essential role of NSP6 for viral survival and identifies this protein as a key molecular target for viral evolutionary surveillance.

## 4. Insights from Antiviral Research Targeting NSP6

As a highly conserved and functionally essential core protein throughout the coronavirus life cycle, NSP6 features high sequence conservation, low propensity for drug resistance and indispensable biological functions. These properties make it an ideal target for the development of broad-spectrum anti-coronavirus drugs. Currently, drug discovery targeting NSP6 has advanced in multiple directions, with therapeutic strategies designed to interfere with its core biological functions.

### 4.1. Inhibitors Targeting DMV Formation

K22 is a well-characterized inhibitor that targets NSP6-mediated formation of viral replication organelles [68]. It exerts antiviral activity by acting on NSP6. Mutations in NSP6 can directly attenuate the inhibitory efficacy of K22, which constitutes the primary mechanism underlying viral drug resistance. Studies have verified that K22 directly binds to coronavirus transmembrane proteins, with NSP6 as its principal target. By disrupting the ER zipper activity of NSP6, K22 blocks DMV biogenesis and ultimately suppresses viral replication.

Under the selective pressure of K22, specific point mutations arise within the TM1-TM3 regions of the NSP6 transmembrane domain. These mutations alter the spatial conformation of NSP6 and disrupt its binding pocket for K22, thereby preventing effective drug binding. This process leads to the emergence of K22 resistance and markedly reduces or even completely abolishes the inhibitory effect [76]. Naturally occurring adaptive mutations in NSP6 of coronaviruses, including SARS-CoV-2, such as the ΔSGF and ΔLSG deletions, moderately reduce viral susceptibility to K22 independent of drug selection and further weaken its antiviral potency [30].

Notably, NSP6 mutations conferring K22 resistance impair the intrinsic functions of NSP6. Consequently, viruses exhibit reduced DMV production and aberrant DMV architecture, accompanied by pronounced declines in replication efficiency, infectivity and viral titer. In short, viruses acquire drug resistance at the cost of replicative fitness. Furthermore, K22 exhibits broad-spectrum inhibitory activity against multiple coronaviruses across the Alpha, Beta and Gamma genera, such as SARS-CoV-2, MHV, IBV and HCoV-229E. Given that K22 targets the highly conserved transmembrane domains of NSP6, viral resistance is less likely to develop. Therefore, K22 represents a promising lead compound for the development of broad-spectrum anti-coronavirus therapeutics.

### 4.2. Other Potential Targeting Strategies

Given the essential functions of NSP6, the ERAD-lipid metabolism and autophagy-lysosome pathways modulated by this protein are also regarded as potential targets for pharmacological intervention [20,21]. In theory, inhibitors targeting the ERAD pathway or lipolysis can block NSP6-induced hijacking of lipid metabolism and cut off lipid supplies for DMV biogenesis. Compounds that restore autophagic flux are able to recover host innate immune responses and counteract NSP6-mediated immune evasion. Nevertheless, these strategies remain purely theoretical based on current mechanistic findings. To date, no NSP6-specific inhibitors have been experimentally validated, and relevant research is still in the exploratory stage.

### 4.3. Structure-Based Drug Design

With advances in cryo-electron microscopy and AlphaFold structure prediction [46], the topology and transmembrane conformation of NSP6 have been gradually elucidated, laying a foundation for structure-based drug design. Approaches including molecular docking and virtual screening can be applied to target functionally critical sites of NSP6, such as its conserved transmembrane regions and dimerization interfaces, so as to screen potential small-molecule inhibitors and build up candidate compounds for subsequent drug development. However, none of the compounds obtained from virtual screening have been validated via in vitro or in vivo assays, and their druggability requires further evaluation.

In conclusion, as a conserved core replication protein of coronaviruses, NSP6 represents a highly promising target for broad-spectrum antiviral drugs. To date, K22 is the only agent proven to exert broad-spectrum antiviral effects by targeting NSP6. Drug development aimed at its roles in metabolic regulation and immune evasion is still in the theoretical exploration phase, calling for further experimental research and clinical translation.

## 5. Conclusions and Future Directions

NSP6 is a multifunctional core factor during coronavirus replication. It participates in ER membrane remodeling, lipid metabolism regulation, autophagy blockade and immune evasion and acts as a crucial hub linking viral replication and host responses. To date, substantial progress has been made in the research of NSP6. In *Betacoronaviruses* such as SARS-CoV-2, the core mechanism by which NSP6 exerts ER zipper activity to drive DMV biogenesis has been well defined. The regulatory network whereby NSP6 hijacks the host ERAD-lipid metabolism pathway to supply lipid materials for DMV membrane expansion has also been systematically elucidated. Accumulating evidence confirms that NSP6 antagonizes host innate immunity by inhibiting the TBK1-IRF3 signaling axis and promoting STING1 degradation. Furthermore, the modulatory effects of adaptive mutations including ΔSGF and ΔLSG on viral replication and immune evasion have been characterized, providing molecular markers for viral evolutionary surveillance.

Nevertheless, several critical scientific questions remain to be addressed. The high-resolution structure of NSP6 and its complex with NSP3/NSP4 have not yet been solved. Current structural information of NSP6 is mainly derived from AI prediction tools such as AlphaFold. Its full three-dimensional conformation, dynamic changes in transmembrane regions, and the architecture of the NSP6-NSP3-NSP4 complex during replication organelle assembly lack precise characterization. These gaps hinder in-depth mechanistic insights into membrane remodeling and the advancement of structure-based drug design. The dynamic interaction network between NSP6 and host factors also requires systematic elucidation. Although partial interactions between NSP6 and ERAD components, autophagy-related proteins and immune signaling molecules have been identified, further studies are needed to clarify the temporal interaction networks of NSP6 throughout the viral life cycle, the regulatory mechanisms of host factors on NSP6 functions, and the divergence of host interactions among NSP6 homologs from different coronavirus genera.

In vivo effects of mutations on viral pathogenicity and transmissibility need to be verified using animal models. For instance, the gain-of-function effects of mutations such as ΔSGF are primarily validated in cell-based assays. Their impacts on in vivo replication efficiency, tissue tropism and pathogenicity remain to be further examined using animal models, including humanized mice. Moreover, the synergistic effects of distinct NSP6 mutations and the mechanisms underlying selective pressure during viral evolution require thorough investigation.

In addition, safe, potent and broad-spectrum drugs targeting NSP6 are still in demand. To date, K22 is the only compound confirmed to exert broad-spectrum antiviral activity via NSP6 targeting. Specific inhibitors against NSP6-regulated ERAD-lipid metabolism, the autophagy-lysosome pathway and immune evasion are still at the theoretical stage. No candidate compounds have been validated by in vitro and in vivo experiments, and their druggability, safety and potential for clinical translation await comprehensive evaluation.

With the progressive elucidation of the functional mechanisms of NSP6, its high conservation and essential roles establish it as an ideal target for the development of broad-spectrum anti-coronavirus drugs. Future studies should further clarify the functional differences in NSP6 across distinct coronavirus genera, resolve its full-length structure and comprehensive protein interaction networks, and advance the research, development and clinical translation of NSP6-targeted broad-spectrum antivirals. These efforts will provide novel strategies for the prevention and control of emerging and re-emerging coronavirus infections. Collectively, the NSP6-targeted broad-spectrum antiviral strategy holds great promise as a vital tool to tackle future coronavirus outbreaks.

## Figures and Tables

**Table 1:** Biological functions and potential antiviral target sites of coronavirus NSP6.

Functions	Potential Antiviral Target Sites	Reference
Participation in the formation of the DMV	TM1-TM6 transmembrane regions, AH amphiphilic helices, and NSP3/4 binding interface	[24]
Regulation in lipid metabolism and the ERAD pathway	Lipid droplet anchor localization site	[30]
	ERAD-related protein binding domain	[20]
Dual regulation of autophagy	Beclin1 binding site	[33]
	MLN1/ATP6AP1 interaction interface	[21,34]
Antagonization in the innate immune response	TBK1/IKKε binding pocket	[35]

## Data Availability

No new data were created or analyzed in this study. Data sharing is not applicable to this article.
